# Identifying Cognitive Mechanism Underlying Situation Awareness of Pilots’ Unsafe Behaviors Using Quantitative Modeling

**DOI:** 10.3390/ijerph18063052

**Published:** 2021-03-16

**Authors:** Shaoqi Jiang, Weijiong Chen, Yutao Kang, Jiahao Liu, Wanglai Kuang

**Affiliations:** 1 College of Ocean Science and Engineering, Shanghai Maritime University, Shanghai 201306, China; sqjiang95@163.com (S.J.); wjchen57@163.com (W.C.); jiahao@shmtu.edu.cn (J.L.); 2 Merchant Marine College, Shanghai Maritime University, Shanghai 201306, China; k1925658211@126.com

**Keywords:** ship pilots, unsafe behaviors, situation awareness, theory of planned behavior, structural equation modeling

## Abstract

Situation awareness (SA) of pilots’ unsafe behavior can ensure safety onboard. Thus, the cognitive mechanism that controls the SA leading to unsafe behavior must be articulated. This study employs the SA model and theory of planned behavior (TPB) to articulate a quantitative model of ship safe piloting. Firstly, the hierarchical classification framework of unsafe behaviors was constructed as an analytical foundation for rational and unconscious behaviors in sight of cognitive processes, and then the measurement elements of the cognitive mechanisms for behaviors were identified. Subsequently, based on the structural model, a hypothetical model of the cognitive path for unsafe behaviors was proposed by using the extended TPB, where there are four independent variables (i.e., attitude (ATD), subjective norm (SN), and perceived behavioral control (PBC)), one mediating variables (i.e., SA) and two dependent variables (i.e., behavioral intention (BI) and unsafe behaviors (BE)). Finally, this hypothetical model was analyzed with the data resources from extended TPB questionnaire of 295 pilots. Analysis results show that relationships of causation and mediation in the cognitive mechanism are in line with the behavior pattern and SA have a pronounced mediating effect and a strong relevance to the causal chain of extended TPB framework. This study integrated the SA three-level model to understand the motivation–cognition–action–feedback (MCAF) mechanism of pilots’ unsafe behaviors under cognitive mode of information processing through structural model. It would make a valuable contribution to the assessment and intervention of safety behaviors, and provide a basic framework for monitoring the situation awareness of pilot by man-machine interactive measurement technology in the future.

## 1. Introduction

Ship pilotage is an important component of marine traffic safety. With the growth of China’s national economy, the volume of ship pilotage has, over time, become an important measurement of a port’s economic development [1]. The pilots as the main executor of the ship pilotage play a critical role in ensuring traffic safety and environment protection in port waters [2,3]. With statistical analysis of pilotage accidents in Shanghai port, unsafe behaviors accounted for more than 90% in 10 years [4]. Therefore, pilots’ unsafe behaviors as specific representations of the complex man–ship–environment–management system in ship pilotage, the symptomatic problems of behavioral mechanism receive much interest from experts [5].

In order to reduce the incidence of unsafe behaviors, many experts had conducted research on performance evaluation and influencing mechanism identification for unsafe behaviors based on system effectiveness [6]. In term of performance evaluation, it is a direct measurement category that focuses on the effectiveness caused by specific human behaviors in the system, including the behavioral classification, reliability evaluation and prevention measures [7]. For the first time, reason used the human error model to carry out the basic research of reliability assessment, which believed that individual unsafe behaviors are abnormal psychological activities mainly caused by mistakes and violations [8]. Subsequently, Wu et al. [9] proposed an improved reliability and error analysis method based on the evidence theory, which was utilized to evaluate the probability of human error in the process of accident causation.

On the other hand, the identification of influencing mechanism is an external architectural study that explored the correlated mechanism of behaviors in the cognitive processes from the perspective of organization and system, including the influence of factors such as fatigue, experience and safety culture. Representatively, Choo and Grabowski [10] adopted the high reliability organization theory to construct the framework of the effect of safety culture on the specific behaviors, and then verified the positive adjustment function of safety culture through the questionnaire of 1169 crews. In general, performance evaluation was an important reference way to quantify unsafe behaviors, confirmed by the study on the identification of influencing mechanism from the dimension of organization and system. However, the causal relationship between cognitive factors that is the essential mechanism of unsafe behavior has not been consistently highlighted.

Through the literature review, it is shown that the true causal and associative relationship between unsafe behaviors and situation awareness (SA) of ship pilots has not been investigated in a modeling format. Moreover, the applicability of theory of planned behavior (TPB) formwork to pilot’s rational behaviors, and the role of SA as a mediator in the relationship between TPB variables and pilots’ unsafe behaviors based on cognitive perspective have not been explored yet. This study aims to confirm the cognitive mechanism of extended TPB framework, which is improved by the SA model, influencing on pilots’ unsafe behaviors using the structural equation numerical model. In this context, hierarchical classification of unsafe behaviors and two questionnaires were designed, including pilots’ unsafe behaviors TPB questionnaire and three-dimension situation awareness rating technology (3D-SART) questionnaire. It not only contributes to identification of important social and individual cognitive paths that affect rational and unconscious unsafe behaviors among ship pilots, but also can help behavior surveillance on accidents prevention systems.

The remainder of this paper is organized as follows. In Section 2, the studies of the behavioral mechanism are reviewed. In Section 3, the structural equation modeling (SEM) [11] combing with hierarchical classification and cognitive causations questionnaires of pilots’ unsafe behaviors is presented. In Section 4, our correlation hypothesis model is confirmed in a specific case, including model application, path dependency analysis and cognitive mechanism identification. In Section 5, the implications for research and practice are discussed. Finally, conclusions are drawn in Section 6.

## 2. Literature Review

The study of unsafe behaviors was derived from marine accidents analysis, which is defined in terms of possible adverse consequences and deviation from system norms [12,13]. Researchers generally focused on the objectivity and specificity of the outcome of the maritime events, and calcified the behaviors including human error that caused the accidents were defined as unsafe behaviors [14,15]. With the continuous development of accident prevention, unsafe behaviors were more regarded as any behaviors that might have a negative effect on the safety of organizations or individuals [16]. The analysis showed that most accidents were due to decision errors and were influenced by physical and mental states, environment, and technology [17,18]. Moreover, the performance of unsafe behaviors was usually associated with risk assessment, in which the features of behaviors were embodied through the expert system and machine learning, such as distance, area, and speed in collision events [19,20]. To quantify the unsafe behaviors, researchers, in part, showed that unsafe behaviors were the outcome of exceeding the acceptable threshold, emphasizing the correlation between behaviors and system standards [21,22]. However, the determination of the standards should be evaluated in combination with the severity of the outcomes of the maritime events, which was empirical subjectivity [23]. In this regard, pilots’ unsafe behaviors were defined in this study as rational or unintentional behaviors that caused or may cause accidents, such as a violation of rules and regulations, lookout negligence, and poor use of good seamanship.

Azjen [24] firstly proposed theory of reasoned action (TRA) that believe human behavior was rational and completely controllable from a cognitive perspective, whereas individual behavior was obviously restricted by management norms and the external environment in the real-word. Thus, the TPB framework based on TRA was constructed, which increased the latent variable of perceived behavioral control (PBC) to develop the new behavioral mode (i.e., attitude (ATD), subjective norm (SN), and PBC influencing behavioral intention (BI); PBC and BI predicting specific unsafe behaviors (BE)) [25]. At present, the predictive function of TPB for social cognitive behaviors in critical safety fields had been effectively verified, but the explanation of latent variables for behaviors was the significant difference [11,26]. Wong and Lee [27] conducted a questionnaire survey based on TPB for workers’ unsafe behaviors in a factory, and the results showed that PBC and SN were the direct predictors of BI, while ATD was a negative prediction. Shaw [28] concluded that ATD and BI were significantly correlated through the study of human factors in aviation maintenance violations.

**Figure 1:**
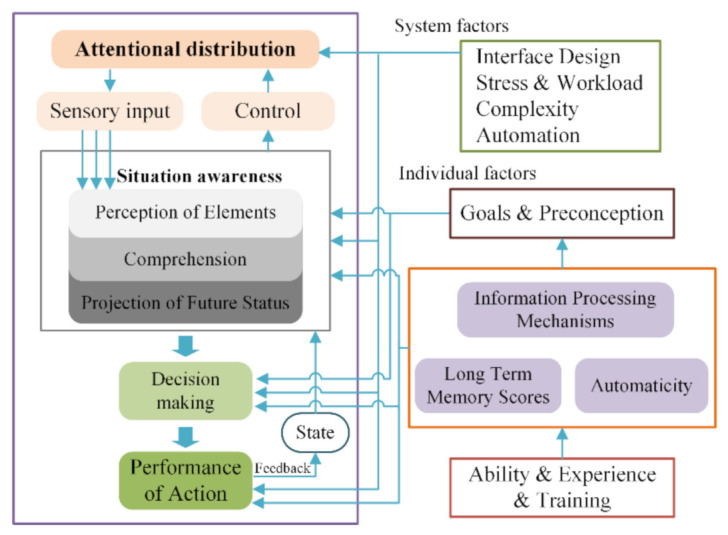
Situation awareness model.

In essential, pilot’s unsafe behavior including social and individual cognition dimensions was rational and unconscious, whereas the initial hypothesis of TPB theoretical framework clarified that human behaviors was completely controlled by individual consciousness [12,29]. Nevertheless, researchers had confirmed that the adoption of the behaviors with high repeatability and experience cannot be completely explained by the rational TPB model [30]. Thus, aiming for better interpretation of rational behaviors, unconscious behaviors, and its correlation function, the situation awareness (SA) variable was introduced to identify the cognitive influencing pathway of individuals. SA was the specific representation of individual cognitive processes, including perception of elements (PB), situational understanding (SU) [31], and prediction of near future states (PS) [32,33], as shown in Figure 1. Hetherington et al. [34] had determined that the deficiency of SA is a directly critical causation of maritime accidents. Grech et al. [35] pointed out 71%, at least in part, of human errors could be attributed to SA-related problems. SA deficiency is a source of human errors that manifested itself as “loss of control over the situation” [36]. Since SA could perceive information through the display of devices and sensory organs, it is vulnerable to the influence of individual and social factors, such as experience, personal expectations, workload, and so on [37]. Therefore, the variables in the TPB framework (i.e., PBC, SN, and ATD) could realize the correlation of cognitive functions by influencing the information perception, understanding, and perception of SA (Figure 1).

Within the complex system of behavioral cognition, the research of unsafe behavioral control is mainly concerned with human reliability analysis (HRA). Patriarca et al. [38] used the multimethod approach to build an intelligent framework with identifying current research streams and outlining the potential for future research in the HRA fields. In maritime industries, the skills–rules–knowledge (SRK) framework attempted to introduce cognitive concepts for effectively distinguishing behavioral performance, which was a traditional application method for HRA quantification combining with expert system. Furthermore, human factor analysis and classification system (HFACS) began to systematically construct the influencing framework of behavioral performance based on the organizational management level, but without consideration of the internal influencing on individual cognitive mechanism [39]. Therefore, the SA model combines individual and external cognitive influencing factors to construct behavioral cognitive model, which provides the possibility for the quantification of the cognitive process.

For quantifying the relationship between latent cognitive variables in the extend TPB formwork, SEM was proposed to analyze the interaction between structural variables based on the covariance matrix of variables [40]. This method integrated multiple regression analysis, path analysis, and confirmatory factor analysis, and considered the influence of measurement errors, and was not restricted by the assumptions of path dependency. The core of SEM is to estimate all the parameters of variables simultaneously with the maximum likelihood method, considering the fitting degree of the model as a whole by measuring the difference between the covariance of the theoretical model and the actual covariance [41]. McBride, Carter, and Phillips [32] used the SEM method to explore the intention of unsafe behaviors through TPB in conjunction with psychosocial factors when driving among young drivers.

In a word, TPB is a social psychological model that explores the mechanism of rational behavior, including attitude, subjective norms, perceived behavioral control, and behavioral intention variables [32,42]. As a significant predictor of behaviors, behavioral intention had been effectively verified in the field of transportation [43]. However, due to the complexity and the typical empirical properties on the ship pilotage, the unsafe behaviors were the combination of rationality and unconsciousness in the real-word. In this study, the latent variable of SA, as the representation of information processing mechanism in the individual cognitive processes, was introduced to modify the basic TPB model. Subsequently, the structural equation model of pilots’ unsafe behaviors was constructed to identify path dependency from a cognitive correlation perspective.

## 3. Research Methods and Models

**Figure 2:**
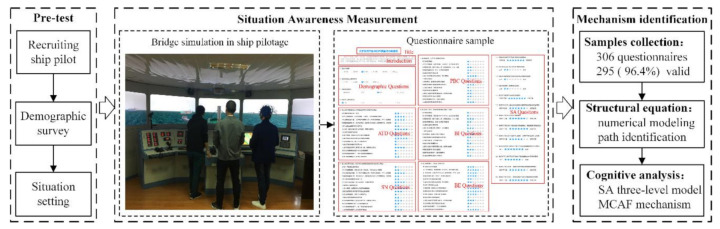
Research framework.

This study focused on investigating the cognitive mechanism of unsafe behaviors among ship pilots, as shown in Figure 2. For the interpretation of rational behaviors, unconscious behaviors and its correlation function in ship pilotage, the TPB model should be improved from two aspects, namely behavioral cognition and model interpretation. First, the improvement of behavioral cognition was reflected in the hierarchical classification of pilots’ unsafe behaviors. Additionally, then the questionnaires based on the extended TPB formwork were formulated, which was developed by the SA model to identify the crucial influence factors and pathways on the unsafe behaviors through multivariate numerical modeling. Subsequently, the SA three-level model was integrated to construct the motivation–cognition–action–feedback (MCAF) mechanism.

### 3.1. Hierarchical Classification of Unsafe Behaviors

**Figure 3:**
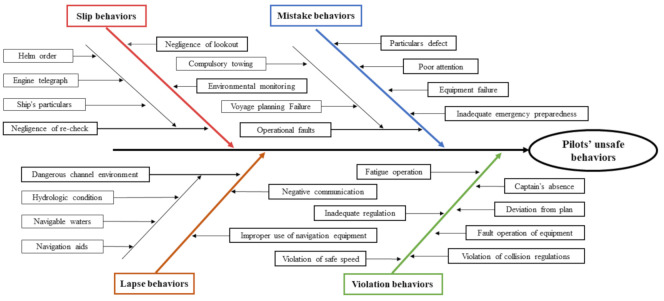
Pilots’ unsafe behaviors hierarchical classification framework.

At present, the behavioral classification framework based on SRK was heavily adopted, which was the attempt to introduce cognitive factors into behavioral research [44]. With the development of the theory of cognitive psychology, the information processing of behavioral cognition gradually formed two modes: “controlled or conscious processing” and “automatic or unconscious processing”, playing an associated role in the real-world [45]. During the ship pilotage, according to the rating scales on hazardous events and occurrence probability formulated by Darbra et al. [46], Chen [47] preliminarily determined the measurement items related to pilots’ unsafe behaviors combining with HFACS. It could be classified into slip (SB), lapse (LB), violation (VB), and mistake (MB). For the purpose of isotropy analysis and implicit rating of questionnaire variables, the rational and unconscious pilots’ unsafe behaviors categories were confirmed (e.g., violation of pilot regulations, negative communication with related personnel and fatigue operation). Pilots’ unsafe behaviors hierarchical classification framework is shown in Figure 3.

### 3.2. TPB Structural Measurement

To explore the correlation between TPB structural variables and SA, this study combined expert experience and incident reports to adapt the TPB and SA scale. All variables were measured by a 7-level Likert scale to identify the influencing mechanism of social cognition and situational cognition on pilots’ unsafe behaviors. In detail, the TPB structural scales listed in Table 1 were adapted from the study of Douglas et al. [48] in vehicle driving, which have been reviewed by senior experts of marine technology including 10 safety engineering and management, 15 maritime supervision, and 12 senior pilot experts. They ranged from 40 to 55 years old, and had 15.3 years of experience in management and pilotage on average. Moreover, the existing measuring items were cross validated by the vote integration mechanism based on the majority rule. In detail, the basic measurement items were obtained through a literature review, and the preliminary adjustment was carried out through the expert system. After being summarized, the measurement items with more than two-thirds of the votes were retained by independent voting. Finally, a total of 15 unsafe behaviors were measured based on SB, LB, VB, and MB. The measurement items of the TPB structure were designed respectively from the dimensions of ATD, SN, PBC, and BI based on four categories of specific unsafe behaviors, as shown in Table 2, in which the “No.” indicated the number of measurement items of the questionnaire. Furthermore, the measurement of SA variable of the SEM was shown in Table 3 of Section 3.3 through 3D-SART.

### 3.3. Situation Awareness Measurement

**Figure 4:**
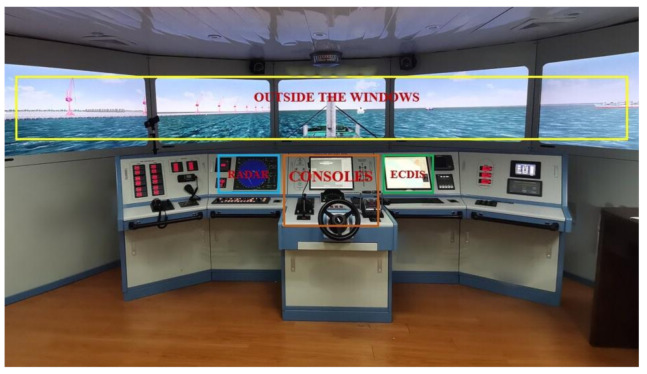
The experimental situation in ship pilotage.

As shown in Figure 4, all situations involving pilotage tasks were selected from the database of qualification examinations, where the subject was Shanghai Yangshan port outbound. The measurement of SA was implemented by the measurement framework of SA level, which consists of three dimensions and ten subdimensions as shown in Figure 3 [49]. Since the non-intrusive and ease of implementation, 3D-SART was one of the most representative methods in the self-rating techniques for measuring the SA level [50]. It was developed from three dimensions including operators’ attentional demands, attentional supply and situational understanding, then ten subdimensions were constructed as the measurement items combining with practical pilotage situation. The results were calculated from equation that SA score = understanding−(demand−supply). Besides, 7-level Likert measurement was adopted in all items. Furthermore, 3D-SART was used to measure the pilot’s SA level before and after the experiment. The score before the experiment was used as a reference to ensure that real levels in the pilotage experiment are obtained, that is, under the premise that the SA score before the experiment was in the normal range, the SA score after the experiment was the actual SA level. Otherwise, the outlier data was deleted.

### 3.4. Theoretical and Hypothesis Development

In TPB structural variables, PBC referred to the perception of the difficulty of behaviors’ implementation by oneself, reflecting the role of experience and expectation. SN manifested the behaviors influenced by social pressures, including morality and family. ATD was defined as a positive or negative assessment of the individual’s behaviors being discussed. BI as the preposition of the behavior was the direct determinant of the actual behaviors, which showed the possibility and subjectivity of the individual to adopt specific behaviors. Since the TPB model had been widely verified in the field of behavior research, based on the conclusions of PBC, SN, and ATD respectively in the critical safety field, and combined with the piloting operation process, the following hypotheses were made:

**Hypothesis** **1** **(H1).** *PBC has a significant effect on BI.*

**Hypothesis** **2** **(H2).** *SN has a significant effect on BI.*

**Hypothesis** **3** **(H3).** *ATD has a significant effect on BI.*

**Hypothesis** **4** **(H4).** *BI has a significant effect on BE.*

As a critical risky cognitive element, SA had been widely recognized in the maritime industries [51,52]. With the continuous development of the theoretical model of SA, the specific structure and influencing mechanism had begun to be explored in ship pilotage [53]. The Endsley’s three-level model separated the operator’s behavioral cognitive outcomes and processes by three levels of perceiving, understanding, and predicting, and integrated the influences of internal and external factors on each level into the construction of model, which laid an important theoretical foundation for exploring the influencing mechanism of SA on unsafe behaviors [23]. Thus, the following hypotheses were made:

**Hypothesis** **5** **(H5).** *SA has a positively correlated with BI.*

**Hypothesis** **6** **(H6).** *SA has a positively correlated with BE.*

### 3.5. Structural Equation Numerical Modeling

The SEM is composed of the measurement model and structural model. The measurement model investigates the covariation effect between latent variables and observed variables. The equation matrices of measurement model are shown as follows:

X = Λ_x_ ξ + δ (1)

Y = Λ_y_ η + ε (2)

where among them,

Λ_x_: Coefficient matrixes between measurement variables and exogenous latent variables;

Λ_y_: Coefficient matrixes between measurement variables and endogenous latent variables;

X: Measurement variable matrixes of exogenous latent variables;

Y: Measurement variable matrixes of endogenous latent variables;

ξ: Exogenous latent variable matrixes;

η: Endogenous latent variable matrixes;

δ: Residual matrixes of endogenous latent variables;

ε: Residual matrixes of exogenous latent variables.

The structural model examines the correlation between latent variables and observed variables. The equation matrices of structural model are shown as follows:

H = βη + Γξ + ζ(3)

where among them,

β: Coefficient matrixes between endogenous latent variables;

Γ: Coefficient matrixes between exogenous latent variables;

ζ: Residual items of random disturbance, namely the endogenous latent variable was not interpreted part in the SEM.

**Figure 5:**
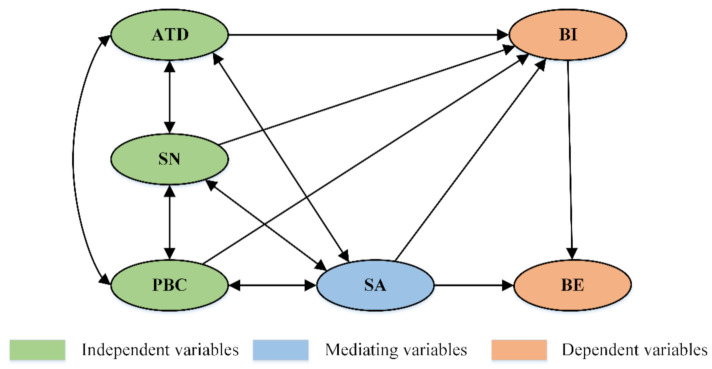
Path of the hypothetical structural equation model.

In this study, the hypothesis structure model of pilots’ unsafe behaviors is thus confirmed, as shown in Figure 5. The category I factors of the TPB framework and SA are used as latent variables (indicated by ellipses), and the corresponding category II factors of measurement items are used as observation variables (indicated by boxes).

## 4. Case Study

### 4.1. Questionnaire Samples Acquisition and Correlation Analysis

**Figure 6:**
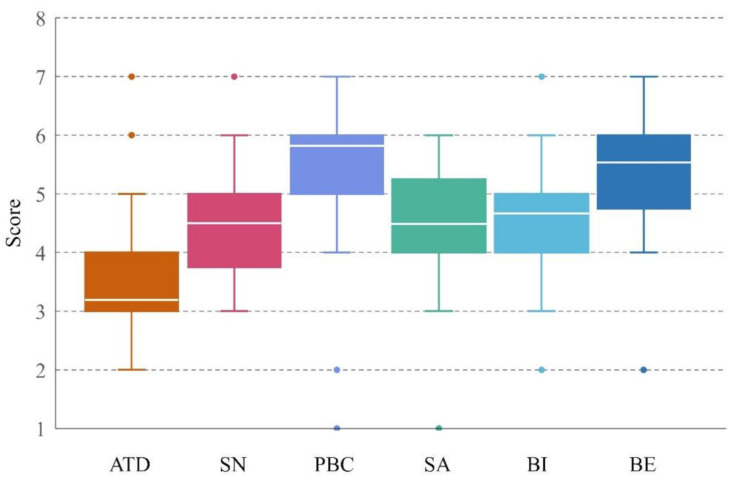
Box whisker plot of the questionnaire samples.

In order to enable the fitting of the questionnaire data into the hypothesis model, collected behavioral factors were quantified according to the social and individual cognitive path of the impact on the pilots’ unsafe behaviors. In this paper, a total of 306 questionnaire samples were sent to the pilot station of Shanghai Port, 295 samples were recovered with an effective rate of 96.4%, meeting the requirements of statistical analysis. To evaluate and synthesize the measurement variables, a workshop was conducted with subject-matter experts in cognitive analysis of unsafe behaviors. All the measurement items in the questionnaires were formatted to numerical analysis data, which was simplified and reserved by ATD, PBC, SN, SA, BI, and BE (Figure 6). The dispersion of data in different quartiles was basically within the normal range after excluding the abnormal data values, i.e., these dots in Figure 6. The Cronbach’s of each latent variable was thus greater than 0.7 between 0.776 and 0.885, and the combined reliability (CR) was greater than 0.88, indicating that the structural equation model had good reliability.

In order to ensure the explanatory ability of latent variables for pilots’ unsafe behaviors, significant correlation paths were identified by the confirmatory factor analysis (CFA). The average variance extracted (AVE) (greater than 0.5) and the square root of AVE (greater than the correlation coefficient of other different latent variables) were effective indexes that verify the convergence and discriminant validity of the model. Furthermore, it was found that the adjusted endogenous latent variables had a better performance as shown in Table 4. In addition, combined with the standardized path coefficient and its significance analysis, effective correlation modes were obtained as shown in Table 5. As for the measurement model, the correlation coefficient of cognitive paths reached a significant level, and the correlation coefficient of SA and PBC was the largest, indicating that the individual cognitive processes and self-control ability of unsafe behaviors influenced each other. However, SN had no significant correlation with SA and ATD, respectively. This result in line with the reality, that is, the experiential properties of individual cognition on behaviors during ship pilotage made pilots less susceptible to the influence of social norms.

### 4.2. Parameter Estimation

The goodness-of-fit index of the amended model was shown in Table 6, which was evident that each index was within a reasonable adaptation standard. It was indicated that the model fit well and could be used for subsequent path identification and analysis of the latent variable.

### 4.3. Path Dependency Analysis

**Figure 7:**
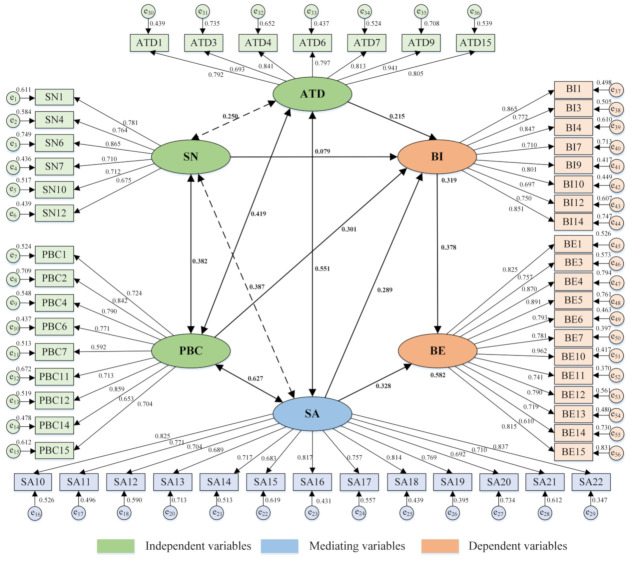
Cognitive path dependency of pilots’ unsafe behaviors.

The cognitive moderating model of pilots’ unsafe behaviors (Figure 7) could be constructed by the path dependency analysis of the research hypothesis and the estimated result of the path influencing coefficient (Table 7). Figure 5 showed that the mediate effect of BI between PBC, SN, ATD, and BE had been effectively verified in ship pilotage. Firstly, the path coefficient of SN and ATD on BI was relatively low, indicating that ship pilotage emphasized the importance of individual roles and the experiential properties. Secondly, PBC implied the interaction between individual and social cognition, and had a directly significant effect on BI from the perspective of self-control ability. It was clarified that pilots often made decisions after taking the initiative to evaluate the difficulty of behaviors in real-word. Subsequently, two paths were not significant (dashed in Figure 7): ATD correlated with SN and SA correlated with SN. For this reason, these two paths were removed to improve the cognitive mechanism model. Finally, SA that is an extended variable at the individual cognitive level in the TPB framework produced indirect and direct effects on both BI and BE with a high significance level in the path dependence analysis. This result proved that the perception, understanding, and prediction mechanism based on information processing progresses could effectively explain the specific cognitive process of pilots’ unsafe behaviors.

The results of path dependence analysis could generalize the cognitive process of pilots’ unsafe behaviors as a self-stress response with experiential cognitive procedures in a specific situation, and it was influenced by social cognitive factors including SN, PBC, and ATD. With regard to the level of social cognition, PBC had the greatest influencing coefficient on the path of BI (0.301), indicating that the pilots might actively evaluate the difficulty of unsafe behaviors during the implemental processes. Additionally, as a specific representation of individual cognitive processes, SA had direct (0.328) and indirect (0.289) effects on endogenous and exogenous latent variables, and was significantly correlated with PBC (0.627, *p* < 0.01) and ATD (0.551, *p* < 0.01). It implied that there was a mediate effect in the influence path of SA on behavioral intention and unsafe behaviors.

### 4.4. Cognitive Mechanism Identification

**Figure 8:**
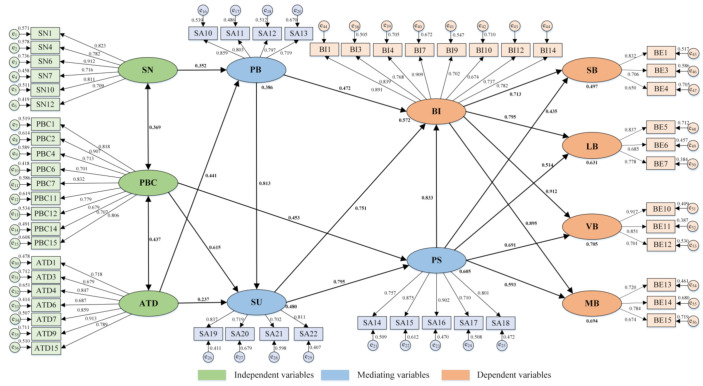
Structural model of the cognitive mechanism.

Cognitive mechanism recognition was used to integrate social cognitive and individual cognitive factors to reveal the cognitive processes of rational and unconscious unsafe behaviors. The mediating role of SA in the path dependency analysis had been effectively verified. Therefore, according to the hierarchical classification of behavior categories and the three-level model, the structural model was optimized to clarify the influence of the structural contents of SA on specific unsafe behaviors. The structural model of observed variables with goodness-of-fit (Table 8) was determined in the structural equation numerical model by combining the questionnaire data (Figure 8). This result showed that SN and ATD significantly had an effect on PB (0.352, 0.441), while ATD also making a difference to SU (0.237). Additionally, PBC not only had a significant impact on SU (0.615), but also played a significant role in PS (0.453). Furthermore, both unconscious and rational unsafe behaviors could be effectively explained by SA, and the direct effects on VB (0.691) and MB (0.593) were more significant.

**Figure 9:**
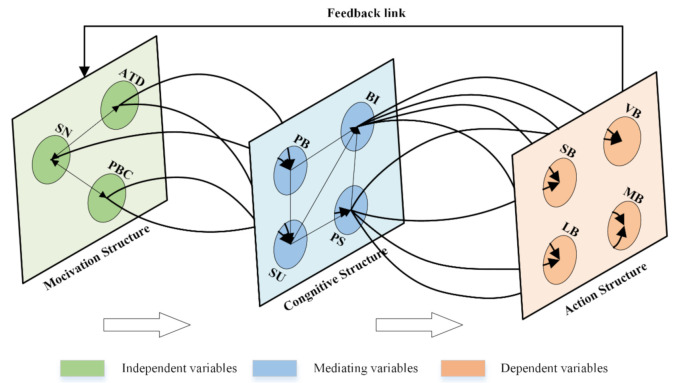
The motivation–cognition–action–feedback (MCAF) cognitive mechanism of pilots’ unsafe behaviors.

In detail, pilots first needed to have a strong motivation to take unsafe behaviors when they had a positive attitude towards special unsafe behaviors of slip, lapse, violation, or mistake, and believed that this behavior could be socially acceptable and easily controlled in a specific situation. The pilots prepared for the construction of mental model, driven by specific motivation, which was manifested that the selective perception was conducive to the positive information of unsafe behaviors, and understanding and prediction of the feasibility in the current situation, thus generating the clear enforceable intention. When such cognitive processes formed fixed experiences or habits, the specific unsafe behaviors were more likely to be taken frequently. Furthermore, the fluke mind and comfortable sensation after the unsafe behavior was adopted would feed back and form a new motivation, prompting the pilot to implement it again. From the above, to realize the embodiment of cognitive mechanism of pilots’ unsafe behaviors, the motivation–cognition–action–feedback (MCAF) mechanism of pilots’ unsafe behaviors was constructed as shown in Figure 9.

## 5. Discussion

Ship pilotage is one of the most hazardous situations in navigation, where there is a disastrous loss of accidents that is mainly caused by unsafe behavior. To identify the cognitive mechanism of pilots’ unsafe behaviors, this study used a structural equation model based on extended TPB questionnaires to explore the influence of cognitive factors and their impact paths on unsafe behaviors. Then, the MCAF cognitive mechanism was constructed composed of the causal and associated effect of cognitive factors on rational and unconscious unsafe behaviors by 295 samples. The findings provide important theoretical foundation for designing interventions to promote pilotage safety. Additionally, the detailed contributions of this research are twofold as follows:

The pilots’ unsafe behaviors include rational and unconscious dimensions, however, the TPB framework is mainly the focus on rational behaviors. Thus, the introduction of potential variables of SA is conducive to the effective identification of specific behavioral cognitive processes. This study added SA variables in the TPB framework to construct a structural equation model based on the integration of explicit and implicit cognition at the social and individual levels. As a result, the TPB as a research framework of rational behavior has been effectively verified in ship pilotage. In detail, the PBC, ATD, and SN variables all had significant direct effects on BI, and BI had the highest path impact coefficient (0.912) on violations (Figure 8). Moreover, SA had significant correlation effect with PBC (0.627, *p* < 0.01), and as a critical mediating variable had significant direct and indirect effect on BI (0.289, *p* < 0.01) and BE (0.328, *p* < 0.01), respectively. In the case of stress and information uncertainty, the recognition-primed decision (RPD) was proposed to effectively explain the process of “bounded rationality” individual’s exploration, judgment, evaluation, and selection of behavioral purpose and implementation path [54]. Based on the findings, the identification pathways of behavioral cognitive processes were complementary to the RPD, which also emphasized the importance of the situational assessment and experience. However, the RPD model is a qualitative description of the decision-making process. In this paper, SA and environmental factors were materialized, and the cognitive influencing path was identified through the situation assessment variables of perception, understanding, and prediction and the environment variables of ATD, PBC, and SN.

With respect to the cognitive mechanism, the SA model realizes the embodiment of cognitive processes of pilots’ unsafe behaviors based on information processing mechanism, including perception, understanding, and prediction. Therefore, the cognitive mechanism of motivation–cognition–action–feedback (MCAF) was constructed in combination with the calculation results of structural equation model to identify the influence path of hierarchical structures of SA cognitive progresses on pilots’ unsafe behaviors. Example verification shows that SA is a mediating factor between the variables of motivation and action structure, where the manifested form of perception, understanding, and prediction conforms to the path dependency mode. The prediction variable of SA structure in the MCAF cognitive mechanism has a strong correlation with the interpretation of the rational and unconscious pilots’ unsafe behaviors.

In application of modern safety science, the identification of behavioral cognitive mechanism at the view of traditional risk and safety provides a good theoretical foundation for the study of resilience engineering at system level. As a new approach of systemic risk management, resilience advocates different agents of man–machine–environment–management system to improve their own and their system’s adaptability in response to the compound disaster and risk, and establishes a risk governance mode covering all-hazard management approach throughout the whole process before, during and after the disaster based on cooperation and organizational learning mechanism [55]. The cognitive mechanism of MCAF considered the individual as a subsystem to conduct research on the behavioral cognitive process. In order to measure with the existing TPB framework, this paper only generalized the contextual factors to the variables of ATD, PBC, and SN. Therefore, subsequent studies will focus on the measurement of contextual factors, and build a resilience governance model for complex pilotage systems based on a comprehensive behavioral cognitive mechanism.

## 6. Conclusions

The cognitive progresses of pilots’ unsafe behaviors are complex, and the influence degree and correlation mode of variables in the cognitive mechanism are different. It is worth noting that the correlation strength of the variables determines the cognitive path of the unsafe behaviors. In this paper, the extended TPB framework, which was developed by SA model, was utilized to research the identification of cognitive mechanism for pilots’ unsafe behaviors during pilotage, based on the structural equation numerical model. The conclusion clarified that ATD and PBC was the strongest factor to PB and SU respectively, and SA had a significant mediating effect on VB and MB. This provides important theoretical support for overall understanding of pilots’ cognitive processes and effective prevention of unsafe behaviors. More specifically, limited resources should be distributed to improve pilots’ ATD and PBC that was effectively positively impacted on the prevention of VB and MB.

There are limitations in the study, but it also associated with future directions. First, SA as a consciousness based on a particular situation should be discussed with the development of subscenes during ship pilotage. Thus, the following experimental procedure could be divided into questionnaire measurement before and after the departure, navigation in fairway, encounter, poor visibility, and anchoring subscenes according to the actual pilot’s competency requirements. Although there are inevitably subjective characteristics in the questionnaire measurement, a starting point is presented for future studies to be conducted in physiological monitoring technology owing to the finding provides the cognitive foundation of unsafe behaviors from pilots’ subjective perspective, which conforms to the experience attribute during actual pilotage work. Therefore, a future study may be conducted the cognitive progresses measurement of PB and SU variables in SA structure among ship pilots using real-time eye-tracking and electroencephalogram monitoring technology, respectively. Moreover, in modern safety science, the resilience governance mode is influenced by the internal and external mechanisms of its complex subsystems, and the behavioral cognitive mechanism at the individual level, as the critical subsystem, lays an important foundation for the establishment of subsequent risk resilience monitoring and early warning model.

## Figures and Tables

**Table 1:** Pilot unsafe behavior classification measurement scale.

Categories	Measurement Items
SB	Occasionally neglect the lookout
	Alcohol may be consumed in small quantities before pilotage
	To hold the situation, while ignoring the implementation of every helm order and engine telegraph
	With extensive piloting experience, communication with relevant personnel can be reduced
LB	Use operations that deviate from normal in emergency
	Occasionally forget the rules of collision avoidance when there is no danger
	Occasionally forget some information about the natural environment in complex waterways
VB	Fatigued piloting for the completion of pilotage
	An opportunity can demonstrate your piloting skills above those of peers
	Try not to use tug for saving cost in vessel arrival and departure
	To improve pilotage efficiency, the nearest route deviating from pilotage plan can be selected
MB	Don’t care too much about safe speed
	Use navigation equipment inappropriately occasionally for some reason
	Leave the bridge briefly to attend to important business
	Due to familiar with pilotage planning and preparation work, need not spend too much energy

**Table 2:** Structure of pilot unsafe behaviors survey scale.

	Variables	Items	Description and Observation Character	No.
Independent	ATD	SB~MB	Understandability of unsafe behavior occurring	5
	SN	SB~MB	Acceptability of colleagues or crews when behaviors are performed	6
	PBC	SB~MB	Difficulty in avoiding unsafe behaviors	7
Dependent	BI	SB~MB	Possibility of attempting unsafe behaviors to complete the pilotage in the near future	8
	BE	SB~MB	Unsafe behaviors have been committed or may be committed	9

**Table 3:** 3D-SART measurement scale in ship pilotage.

Dimensions	Construct	Measurement Items	No.
Attentional demands	Instability of situation	The stability of the traffic environment and vessel in the pilotage (without emergency)	10,11
	Variability of situation	The number of variables, including navigational and environmental elements, should be focused	12
	Complexity of situation	Complexity of the traffic in pilotage waters.	13
Attentional supply	Arousal	The alertness of pilots in pilotage	14
	Spare mental capacity	How much residual energy should be used to deal with emergencies in pilotage	15
	Concentration	Degree of concentration at initial pilotage	16
	Division of attention	The ability to notice multiple information variables simultaneously in pilotage	17,18
Understanding of the situation	Information quantity	Amount of information received and understood	19
	Information quality	Reliability of perceived information	20
	Familiarity	Familiarity of pilotage waters	21,22

**Table 4:** Correlation coefficients and AVE square roots of latent variables.

Variables	AVE	PBC	SN	ATD	SA
PBC	0.594	0.771			
SN	0.648	0.382	0.805		
ATD	0.587	0.419	0.250	0.766	
SA	0.548	0.627	0.387	0.551	0.740

Note: the diagonal is the square root of AVE.

**Table 5:** Correlation characters via adjusted SEM simulation.

Correlation Mode	Estimate	S.E.	C.R.	*p*	Standardized Path Coefficient
PBC ↔ SN	0.311	0.067	4.950	**	0.382
SN ↔ SA	0.288	0.062	4.630	—	0.387
ATD ↔ SA	0.252	0.045	5.634	***	0.551
ATD ↔ PBC	0.219	0.044	5.027	**	0.419
SN ↔ ATD	0.195	0.057	3.419	—	0.250
SA ↔ PBC	0.317	0.052	6.130	***	0.627

*** *p* < 0.01, ** *p* < 0.05.

**Table 6:** Model-fit indices.

Indexes	χ^2^/df	GFI	AGFI	NFI	CFI	TLI	RMSEA
Reference	(1,3)	>0.8	>0.8	>0.9	>0.9	>0.9	<0.08
Estimate value	1.902	0.914	0.883	0.904	0.951	0.941	0.055

Note: χ^2^/df, chi-square and degrees of freedom; GFI, goodness of fit index; AGFI, adjusted goodness of fit index; NFI, normed fit index; CFI, comparative fit index; TLI, Tucker–Lewis index; RMSEA, root mean square error of approximation.

**Table 7:** Model path regression coefficient.

Path Mode	Estimate	S.E.	C.R.	*p*	Path Coefficient	Results
PBC → BI	0.339	0.107	3.172	**	0.301	H1 was found
SN → BI	0.060	0.052	1.150	**	0.079	H2 was found
ATD → BI	0.226	0.084	2.710	**	0.215	H3 was found
BI → BE	0.370	0.096	3.836	***	0.378	H4 was found
SA → BI	0.333	0.123	2.713	***	0.289	H5 was found
SA → BE	0.370	0.105	3.491	***	0.328	H6 was found

*** *p* < 0.01, ** *p* < 0.05.

**Table 8:** After modified model-fit indices.

Indexes	χ^2^/df	GFI	AGFI	NFI	CFI	TLI	RMSEA
Reference	(1,3)	>0.8	>0.8	>0.9	>0.9	>0.9	<0.08
Estimate value	1.592	0.925	0.897	0.907	0.938	0.945	0.043

## Data Availability

This study did not report any data.

## References

[1] Wang L., Liu Q., Dong S.Y., Soares C.G. (2019). Effectiveness assessment of ship navigation safety countermeasures using fuzzy cognitive maps. Saf. Sci..

[2] Main L.C., Wolkow A., Chambers T.P. (2017). Quantifying the Physiological Stress Response to Simulated Maritime Pilotage Tasks: The Influence of Task Complexity and Pilot Experience. J. Occup. Env. Med..

[3] Ugurlu O., Kaptan M., Kum S., Yildiz S. (2017). Pilotage services in Turkey; key issues and ideal pilotage. J. Mar. Eng. Technol..

[4] Xue Y.D. (2005). Human Errors and Prevention of Accidents in Ship-pilotage. Navig. China.

[5] Ugurlu O., Yildiz S., Loughney S., Wang J. (2018). Modified human factor analysis and classification system for passenger vessel accidents (HFACS-PV). Ocean Eng..

[6] Luo M.F., Shin S.H. (2019). Half-century research developments in maritime accidents: Future directions. Accid. Anal. Prev..

[7] Petkov G., Todorov V., Takov T., Petrov V., Stoychev K., Vladimirov V., Chukov I. (2004). Safety investigation of team performance in accidents. J. Hazard. Mater..

[8] Reason J. (2000). Human error: Models and management. West. J. Med..

[9] Wu B., Yan X., Wang Y., Soares C.G. (2017). An Evidential Reasoning-Based CREAM to Human Reliability Analysis in Maritime Accident Process. Risk Anal..

[10] Choo A.S., Grabowski M.R. (2018). Linking Workplace Safety to Operational Disruptions: A Moderated Mediation Analysis in Commercial Vessels. J. Bus. Logist..

[11] Ghasemzadeh S., Babazadeh T., Allahverdipour H., Sadeghi-Bazargani H., Kouzekanani K. (2017). Cognitive-behavioral determinants of using helmet by motorcyclists in a rural community. J. Transp. Health.

[12] Lee D., Park J.H., Kim H. (2004). A study on experiment of human behavior for evacuation simulation. Ocean Eng..

[13] Bye R.J., Aalberg A.L. (2018). Maritime navigation accidents and risk indicators: An exploratory statistical analysis using AIS data and accident reports. Reliab. Eng.Syst. Saf..

[14] Youn I.-H., Park D.-J., Yim J.-B. (2019). Analysis of Lookout Activity in a Simulated Environment to Investigate Maritime Accidents Caused by Human Error. Appl. Sci..

[15] Lu C.S., Tsai C.L. (2008). The effects of safety climate on vessel accidents in the container shipping context. Accid. Anal. Prev..

[16] Fam I.M., Nikoomaram H., Soltanian A. (2012). Comparative analysis of creative and classic training methods in health, safety and environment (HSE) participation improvement. J. Loss Prev. Process Ind..

[17] Carotenuto A., Molino I., Fasanaro A.M., Amenta F. (2012). Psychological stress in seafarers: A review. Int. Marit. Health.

[18] Chauvin C., Lardjane S., Morel G.L., Clostermann J.P., Langard B.T. (2013). Human and organisational factors in maritime accidents: Analysis of collisions at sea using the HFACS. Accid. Anal. Prev..

[19] Ozturk U., Birbil S.I., Cicek K. (2019). Evaluating navigational risk of port approach manoeuvrings with expert assessments and machine learning. Ocean Eng..

[20] Vidmar P., Perkovic M., Gucma L., Lazuga K. (2020). Risk Assessment of Moored and Passing Ships. Appl. Sci..

[21] Guo S., Zhou X., Tang B., Gong P. (2020). Exploring the behavioral risk chains of accidents using complex network theory in the construction industry. Phys. A Stat. Mech. Appl..

[22] Rhodes R.E., Courneya K.S. (2005). Threshold assessment of attitude, subjective norm, and perceived behavioral control for predicting exercise intention and behavior. Psychol. Sport Exerc..

[23] Shin M., Lee H.S., Park M., Moon M., Han S. (2014). A system dynamics approach for modeling construction workers’ safety attitudes and behaviors. Accid. Anal. Prev..

[24] Azjen I. (1991). “The theory of planned behavior”, Organizational Behavior and Human Decision Processes, Vol. J. Leis. Res..

[25] Rahman M.M., Lesch M.F., Horrey W.J., Strawderman L. (2017). Assessing the utility of TAM, TPB, and UTAUT for advanced driver assistance systems. Accid. Anal. Prev..

[26] Zhang C.Q., Zhang R., Gan Y., Li D., Rhodes R.E. (2019). Predicting transport-related cycling in Chinese employees using an integration of perceived physical environment and social cognitive factors. Transp. Res. Part F-Traffic Psychol. Behav..

[27] Wong D.B., Lee S.G. (2016). Modelling the predictors of intention in workplace safety compliance of a multi-ethnic workforce. Saf. Sci..

[28] Shaw F.A. (2010). Safety climate and the Theory of Planned Behavior: Towards the prediction of unsafe behavior. Accid. Anal. Prev..

[29] Cheng P.Y., Chu M.C. (2014). Behavioral Factors Affecting Students’ Intentions to Enroll in Business Ethics Courses: A Comparison of the Theory of Planned Behavior and Social Cognitive Theory Using Self-Identity as a Moderator. J. Bus. Ethics.

[30] Brijs K., Daniels S., Brijs T., Wets G. (2011). An experimental approach towards the evaluation of a seat belt campaign with an inside view on the psychology behind seat belt use. Transp. Res. Part F Psychol. Behav..

[31] Sulistyawati K., Wickens C.D., Chui Y.P. (2011). Prediction in Situation Awareness: Confidence Bias and Underlying Cognitive Abilities. Int. J. Aviat. Psychol..

[32] McBride M., Carter L., Phillips B. (2020). Integrating the theory of planned behavior and behavioral attitudes to explore texting among young drivers in the US. Int. J. Inf. Manag..

[33] Raza M.A., Salehi S., Ghazal S., Ybarra V.T., Naqvi S.A.M., Cokely E.T., Teodoriu C. (2019). Situational awareness measurement in a simulation-based training framework for offshore well control operations. J. Loss Prev. Process Ind..

[34] Hetherington C., Flin R., Mearns K. (2006). Safety in shipping: The human element. J. Saf. Res..

[35] Grech M.R., Horberry T., Smith A. (2002). Human Error in Maritime Operations: Analyses of Accident Reports Using the Leximancer Tool. Proceedings of the Human Factors and Ergonomics Society Annual Meeting.

[36] Endsley M.R. (2019). A Systematic Review and Meta-Analysis of Direct Objective Measures of Situation Awareness: A Comparison of SAGAT and SPAM. Hum. Factors J. Hum. Factors Ergon. Soc..

[37] Danial S.N., Smith J., Khan F., Veitch B. (2019). Situation awareness modeling for emergency management on offshore platforms. Hum.–Cent. Comput. Inf. Sci..

[38] Patriarca R., Ramos M., Paltrinieri N., Massaiu S., Costantino F., Di Gravio G., Boring R.L. (2020). Human reliability analysis: Exploring the intellectual structure of a research field. Reliab. Eng. Syst. Saf..

[39] Mazaheri A., Montewka J., Nisula J., Kujala P. (2015). Usability of accident and incident reports for evidence-based risk modeling–A case study on ship grounding reports. Saf. Sci..

[40] Heidari A., Kolahi M., Behravesh N., Ghorbanyon M., Ehsanmansh F., Hashemolhosini N., Zanganeh F. (2018). Youth and sustainable waste management: A SEM approach and extended theory of planned behavior. J. Mater. Cycles Waste Manag..

[41] Menozzi D., Sogari G., Mora C. (2015). Explaining Vegetable Consumption among Young Adults: An Application of the Theory of Planned Behaviour. Nutrients.

[42] Yao X.L., Wu Y.P., Liu H., Zhao X.H., Bian Y., Qu W.N. (2019). Analysis of Psychological Influences on Navigation Use While Driving Based on Extended Theory of Planned Behavior. Transp. Res. Rec..

[43] Cristea M., Gheorghiu A. (2016). Attitude, perceived behavioral control, and intention to adopt risky behaviors. Transp. Res. Part F-Traffic Psychol. Behav..

[44] Rasmussen J. (2012). Skills, rules, and knowledge; signals, signs, and symbols, and other distinctions in human performance models. Ieee Trans. Syst. Man Cybern..

[45] Hunter J., Porter M., Williams B. (2020). Towards a theoretical framework for situational awareness in paramedicine. Saf. Sci..

[46] Darbra R.M., Crawford J.F.E., Haley C.W., Morrison R.J. (2007). Safety culture and hazard risk perception of Australian and New Zealand maritime pilots. Mar. Policy.

[47] Chen S.-T. (2020). An approach of identifying the common human and organisational factors (HOFs) among a group of marine accidents using GRA and HFACS-MA. J. Transp. Saf. Secur..

[48] Douglas M.A., Swartz S.M., Richey R.G., Roberts M.D. (2019). Risky business: Investigating influences on large truck drivers’ safety attitudes and intentions. J. Saf. Res..

[49] Hasanzadeh S., Esmaeili B., Dodd M.D. (2018). Examining the Relationship between Construction Workers’ Visual Attention and Situation Awareness under Fall and Tripping Hazard Conditions: Using Mobile Eye Tracking. J. Constr. Eng. Manag..

[50] Saus E.R., Johnsen B.H., Eid J. (2010). Perceived learning outcome: The relationship between experience, realism and situation awareness during simulator training. Int. Marit. Health.

[51] Man Y., Weber R., Cimbritz J., Lundh M., MacKinnon S.N. (2018). Human factor issues during remote ship monitoring tasks: An ecological lesson for system design in a distributed context. Int. J. Ind. Ergon..

[52] Sandhaland H., Oltedal H.A., Hystad S.W., Eid J. (2017). Effects of leadership style and psychological job demands on situation awareness and the willingness to take a risk: A survey of selected offshore vessels. Saf. Sci..

[53] Mosier K., Fischer U. (2012). Impact of Automation, Task and Context Features on Pilots’ Perception of Human-Automation Interaction. Proceedings of the Human Factors and Ergonomics Society Annual Meeting.

[54] Bond S., Cooper S. (2006). Modelling emergency decisions: Recognition-primed decision making. The literature in relation to an ophthalmic critical incident. J. Clin. Nurs..

[55] Patriarca R., Bergstrom J., Di Gravio G., Costantino F. (2018). Resilience engineering: Current status of the research and future challenges. Saf. Sci..

